# United States Food and Drug Administration’s 510(k) Pathway: Drawing Implications from the Approvals of Brachytherapy Devices

**DOI:** 10.7759/cureus.4230

**Published:** 2019-03-11

**Authors:** Sophie Wang, Albert Manudhane, Harib H Ezaldein, Jeffrey F Scott

**Affiliations:** 1 Dermatology, Case Western Reserve University School of Medicine, Cleveland, USA; 2 Dermatology, Northeast Ohio Medical University College of Medicine, Rootstown, USA; 3 Dermatology, University Hospitals Cleveland Medical Center, Cleveland, USA

**Keywords:** brachytherapy, radiotherapy, medical devices, premarket approval, adverse events, radiation therapy, cancer, keloids, device malfunction

## Abstract

Introduction

Innovations in cancer treatment coupled with an increasing number of cancer patients have led to the growth of brachytherapy devices. The objective of this study is to characterize the development and safety of brachytherapy devices marketed in the United States (US) over the last 15 years.

Methods

We reviewed records from a public US Food and Drug Administration (FDA) database detailing premarket approval of brachytherapy devices. All 510(k) submissions approved between January 1, 2000 and October 31, 2018 were examined. To assess the safety of these devices, we searched the manufacturer and user facility device experience (MAUDE) database for related adverse events.

Results

Twenty-two brachytherapy devices received 510(k) premarket approval, with the first device approved in 2005. Of the 22 devices, 20 (91%) were marketed with specific indications. The most common indications include treatment of skin cancers and keloids (n=7), breast cancer (n=4), and gynecologic/rectal cancers (n=2). A review of the MAUDE database revealed 64 reports of adverse events associated with brachytherapy devices. Common adverse effects include poor device design, use error, and device malfunction that led to the delivery of an inaccurate dose of radiation.

Discussion

Although there are some single-center, short-term studies demonstrating adequate local control and satisfactory cosmesis with brachytherapy, data on long-term outcomes are lacking. Further research is warranted to define appropriate practice guidelines for brachytherapy devices in the treatment of various malignancies.

## Introduction

Radiotherapy, or radiation therapy, is used to eradicate cancer cells, slow tumor growth, and improve quality of life. Approximately 50% of cancer patients may benefit from some form of radiotherapy [1]. Brachytherapy is a subtype of radiotherapy that involves placing the source of radiation within the body to deliver a large dose of radiation directly to the cancer cells. Innovations in cancer treatment coupled with an increasing number of cancer patients has led to the growth of brachytherapy devices on the market. Furthermore, the global radiotherapy devices market is estimated to develop at a compound annual growth rate (CAGR) of more than 6%, and reach at least $7.5 billion by 2022 [2-3].

A 510(k) approval from the Food and Drug Administration (FDA) is required before manufacturers can market a medical device in the United States (US). In order to complete the 510(k) submission, manufacturers must indicate that the device in question must be “substantially equivalent” to a currently approved device. However, clinical evidence documenting safety and efficacy is not required [4]. Given the rise in utility in recent years, we aimed to characterize the development and safety of brachytherapy devices marketed in the U.S. over the last 15 years.

## Materials and methods

In this cross-sectional retrospective analysis, we reviewed records from a public US FDA database detailing premarket approval of devices. All 510(k) submissions for brachytherapy devices were approved between January 1, 2000 and October 31, 2018 and were examined. To assess the safety of these devices, we searched the manufacturer and user facility device experience (MAUDE) database for related adverse events. We received an institutional review board (IRB) exemption for the use of these publicly available databases. Reports were reviewed individually and classified by the number of devices marketed, year of device approval, manufacturing corporations, and indications of use. Descriptive analyses were performed using data visualization software (Tableau Software; Seattle, WA) [5].

## Results

**Figure 1:**
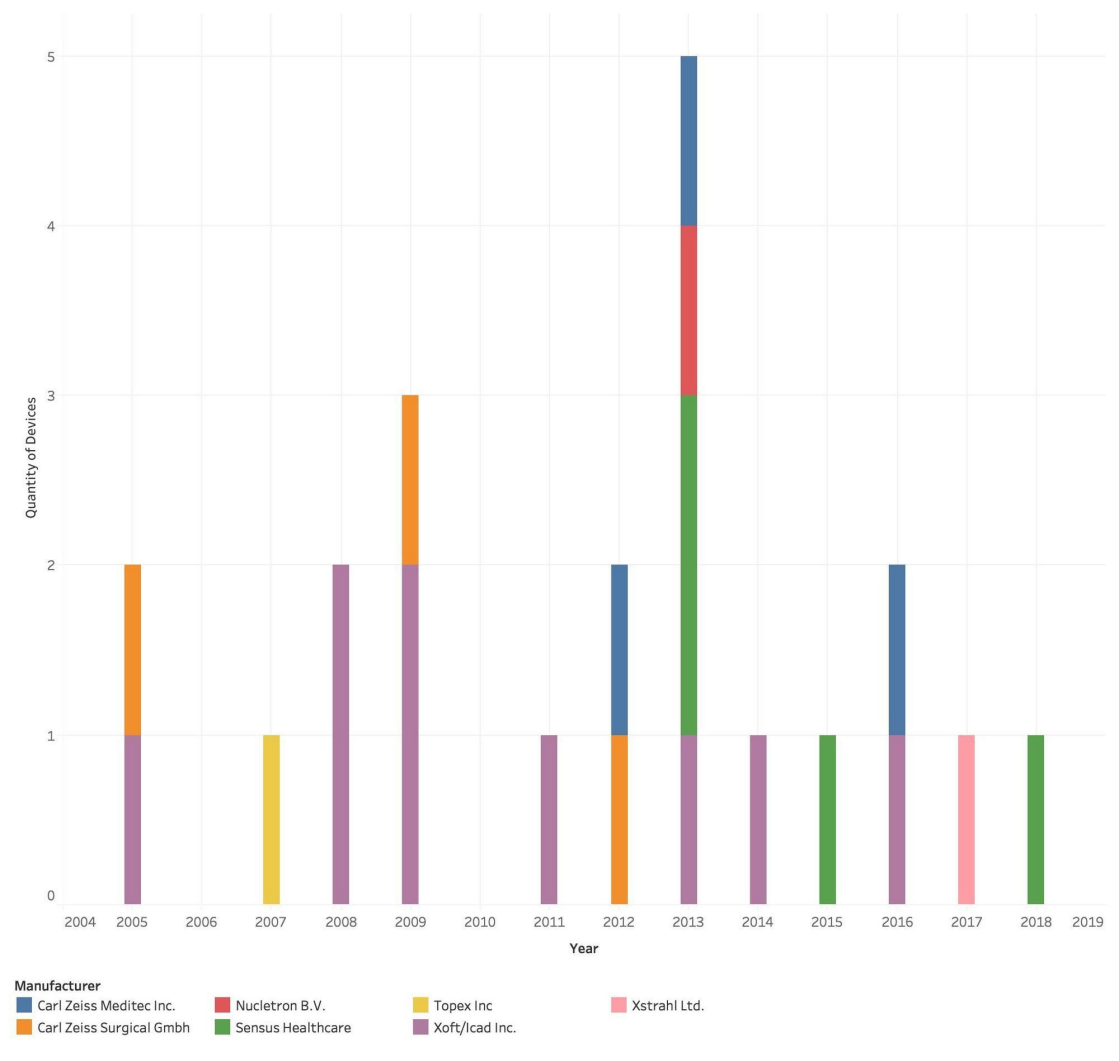
Characteristics of market share by year.

Twenty-two brachytherapy devices received 510(k) premarket approval between 2000 and 2018, with the first device approved in 2005. The number of brachytherapy devices on the market increased since 2005, with the largest number of devices marketed in 2013 (n=5). Xoft Inc. and Carl Zeiss Meditec Inc. were the first two companies to market their devices, both in 2005, and these two companies have produced the greatest number of devices cumulatively from 2005 to 2018 (Figure 1).

**Figure 2:**
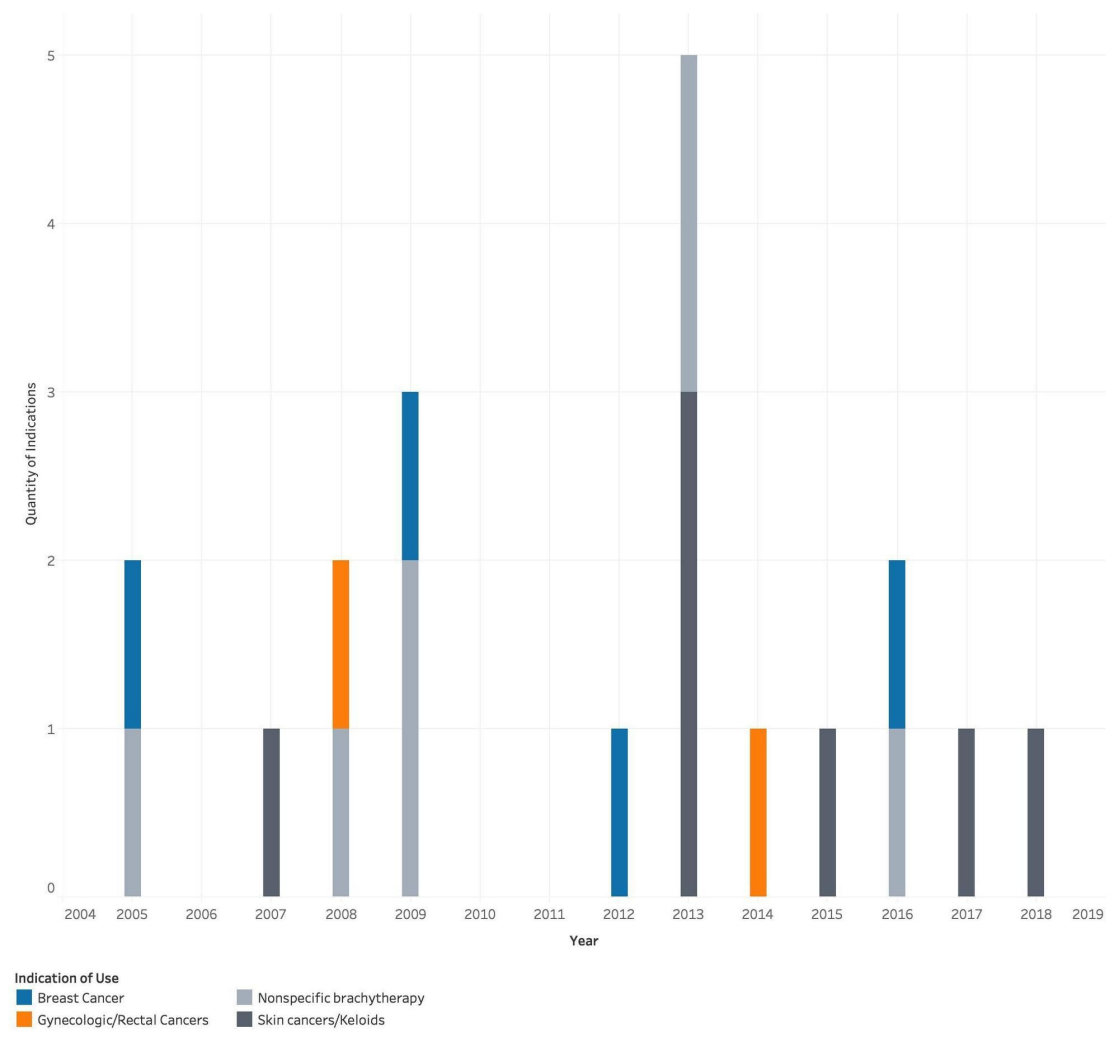
Stated indications of use.

Of the 22 devices, 20 (91%) were marketed with specific indications. The most common indications include treatment of skin cancers and keloids (n=7), breast cancer (n=4), and gynecologic/rectal cancers (n=2). Additionally, some devices did not specify a particular type of malignancy (n=7) (Figure 2). Of note, indications for skin cancer included both high-risk and low-risk malignancies, including basal cell carcinoma (BCC), squamous cell carcinoma (SCC), adnexal carcinoma, Kaposi sarcoma, metatypical carcinoma, Merkel cell carcinoma, lentigo maligna, lentigo maligna melanoma, and cutaneous lymphoma. Gynecologic malignancies included vaginal, uterine, cervical, rectal, and endometrial cancers.

**Figure 3:**
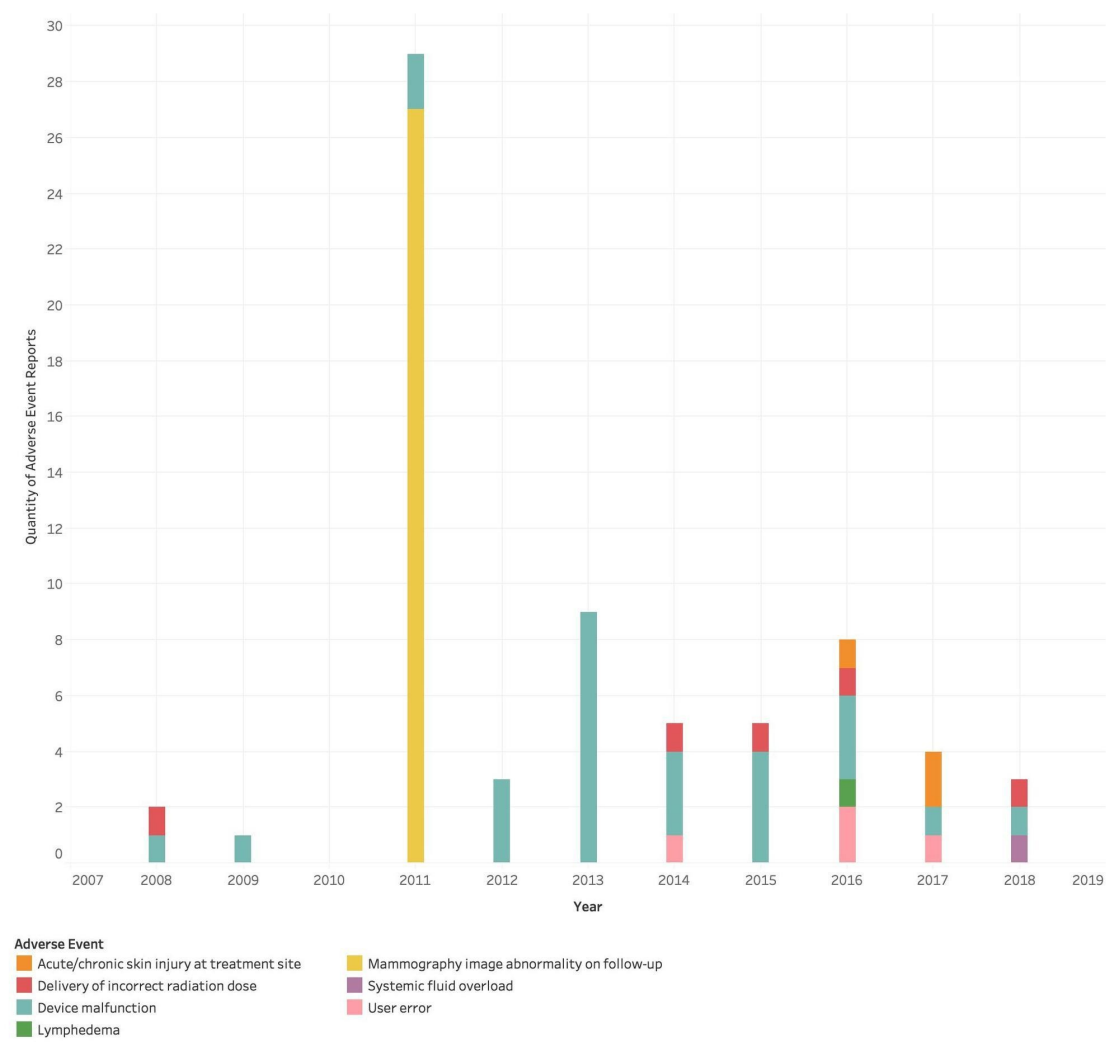
Adverse events associated with brachytherapy devices.

A review of the MAUDE database revealed 64 reports of adverse events associated with brachytherapy devices. Common adverse effects include poor device design, use error, and device malfunction that led to the delivery of an inaccurate dose of radiation. Another adverse effect was discovered on a follow-up mammography, revealing 'innumerable punctate mechanical densities… felt to be artefactual and apparently are debris left from the tungsten shield placed at the time of intraoperative radiation therapy.' Less common adverse effects include wounds at the treatment site, suspected nerve damage, post-inflammatory dyspigmentation, lymphedema, and fluid overload characterized by congestive heart failure and bilateral pleural effusions (Figure 3).

## Discussion

Manufacturers are producing and marketing a greater number of brachytherapy devices as the number of cancer patients receiving radiation therapy increases. Interestingly, these devices have a variety of indications, ranging from the treatment of skin cancer, gynecologic cancer, and breast cancer. Notably, Linos et al. report that brachytherapy marketing targets younger adults and emphasizes 'painless treatment' and good cosmesis for patients 'with busy schedules… not interested in having surgery' [6]. While it is important to consider patient preference and factors such as pain, cosmesis, and lost productivity, it is also necessary to recognize that younger patients with skin cancer may be at highest risk for recurrences and long-term adverse effects related to radiation [6].

Although data on long-term outcomes are not available, a handful of single-center, short-term studies demonstrate that effective local control and satisfactory cosmesis of brachytherapy in treating non-melanoma skin cancers (NMSC) [7]. However, further clinical evidence citing the safety and efficacy of brachytherapy is essential. Additionally, it is crucial that physicians be familiar with appropriate use guidelines as well as indications and contraindications (i.e., skin cancers invading body structures) of brachytherapy. For example, while brachytherapy is recommended for some NMSC, including high-risk tumors such as Kaposi sarcoma and cutaneous lymphoma, it may be less useful for others, such as fibrosarcoma and BCC or SCC of the scrotum [8].

Moreover, while these devices are gaining public attention, adverse events are not well-characterized. We discovered 64 reports of adverse events in the MAUDE database, including delivery of the wrong dose of radiation as well as wounds at the treatment site and lymphedema. The MAUDE database is also marked by significant limitations, including incomplete and unverified data. Thus, the number of adverse events in the MAUDE database may underestimate the true frequency and breadth of complications, and the lack of a universal registry makes determining the true safety profile of these devices challenging. As an example, a study of patients with cardiac implantable electronic devices suggests that patients should follow a total cumulated dose limit and undergo repeated and more frequent follow-up due to potentially dangerous interactions [9].

## Conclusions

A greater number of brachytherapy devices are being manufactured and marketed over the last two decades targeting younger adults who may be drawn to noninvasive treatment options, good cosmesis, and minimal time lost from work. However, adverse events of brachytherapy devices are not well-documented. Although there are some single-center, short-term studies demonstrating adequate local control and satisfactory cosmesis with brachytherapy, data on long-term outcomes are lacking. As such, further research is warranted to define appropriate practice guidelines for brachytherapy devices in the treatment of various malignancies. 
